# Supplementary material for: The Development of CDC25A-Derived Phosphoseryl Peptides That Bind 14-3-3ε with High Affinities
Source: Int J Mol Sci. 2024 Apr 30;25(9):4918. doi: 10.3390/ijms25094918 (PMC11084659; doi:10.3390/ijms25094918)
Supplement: Supplementary file 1 [file ijms-25-04918-s001.zip › ijms-2948345-supplementary.pdf]

Supporting Information

The Development of CDC25A-Derived Phosphoseryl Peptides That Bind 14-3-3ε With High Affinities.

Seraphine Kamayirese, Sibaprasad Maity[†] Laura A. Hansen and Sándor Lovas*

Department of Biomedical Sciences, Creighton University, Omaha, Nebraska 68178, Unites States

[†]Current address: Process Development, Corden Pharma, Boulder, Colorado 80301, United States

Corresponding author:	Sándor Lovas
Address:	Department of Biomedical Sciences<br>Criss II, Room 313<br>Creighton University<br>2500 California Plaza<br>Omaha, NE 68178
Phone:	402-280-5753
Fax:	402-280-2690
E-mail:	slovas@creighton.edu

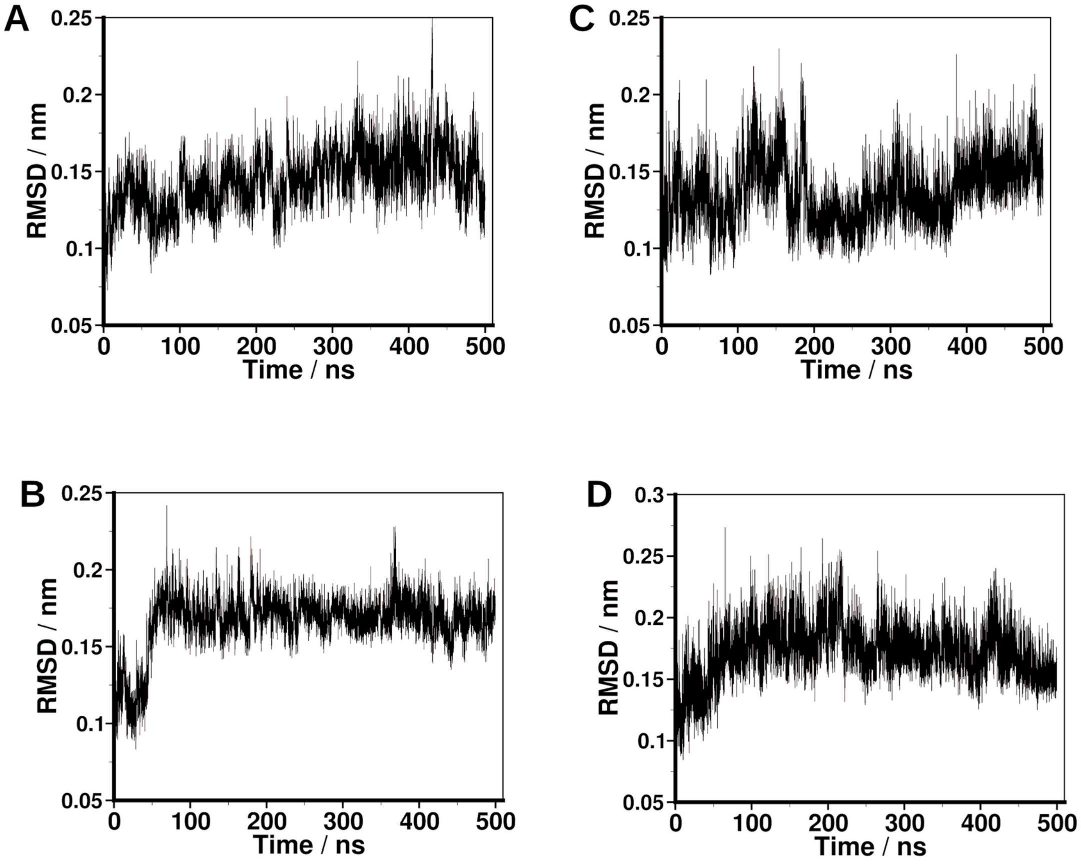

Figure S1. Molecular dynamics simulations of 14-3-3ε in complex with pS analogs of various lengths. Root mean square deviation of system of 14-3-3ε with (A) pS(173-185), (B) pS(173-184), (C) pS(173-183), (D) pS(174-183)

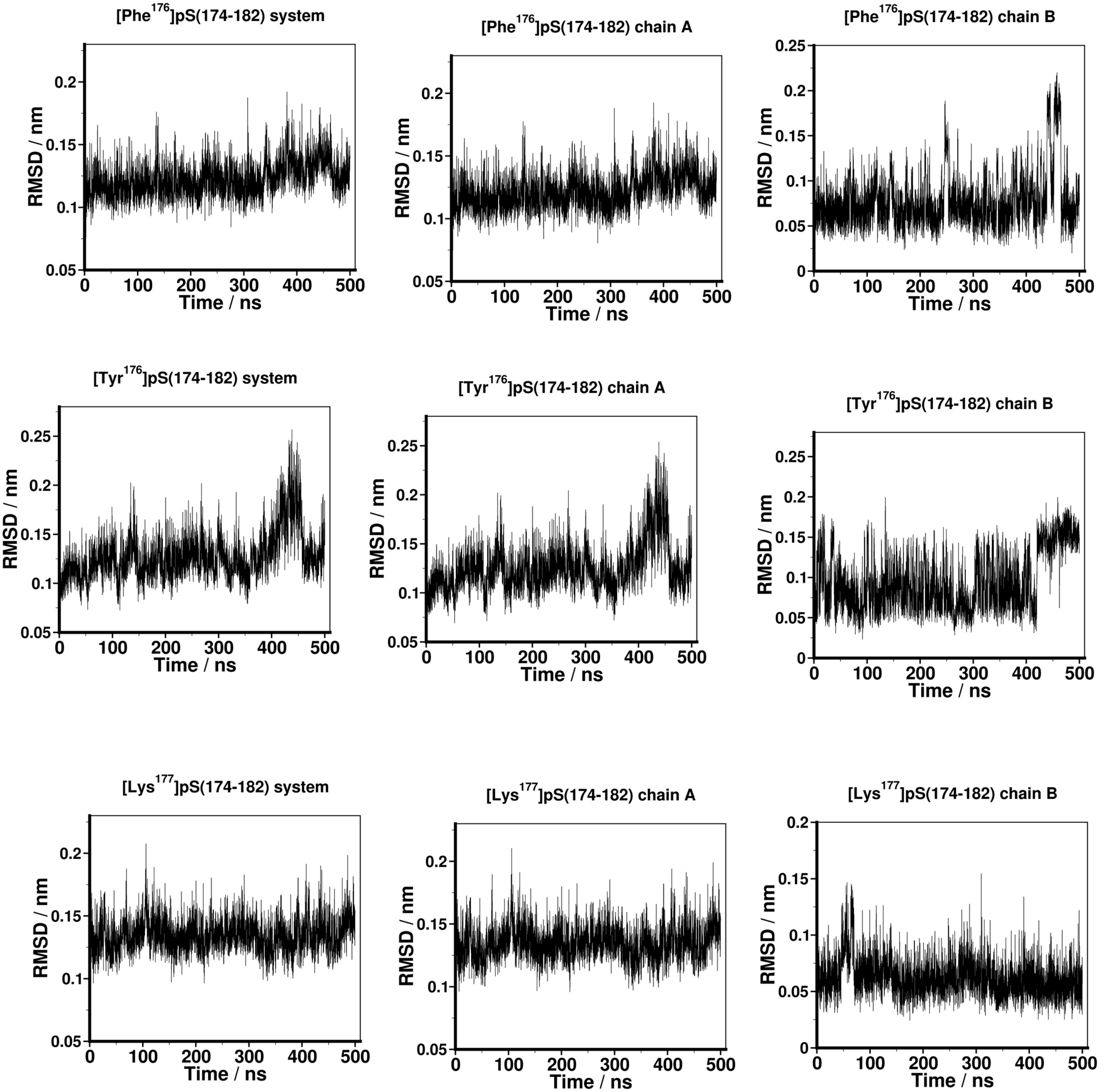
[Phe176]pS(174-182) system
[Phe176]pS(174-182) chain A
[Phe176]pS(174-182) chain B
[Tyr176]pS(174-182) system
[Tyr176]pS(174-182) chain A
[Tyr176]pS(174-182) chain B
[Lys177]pS(174-182) system
[Lys177]pS(174-182) chain A
[Lys177]pS(174-182) chain B
RMSD / nm
Time / ns

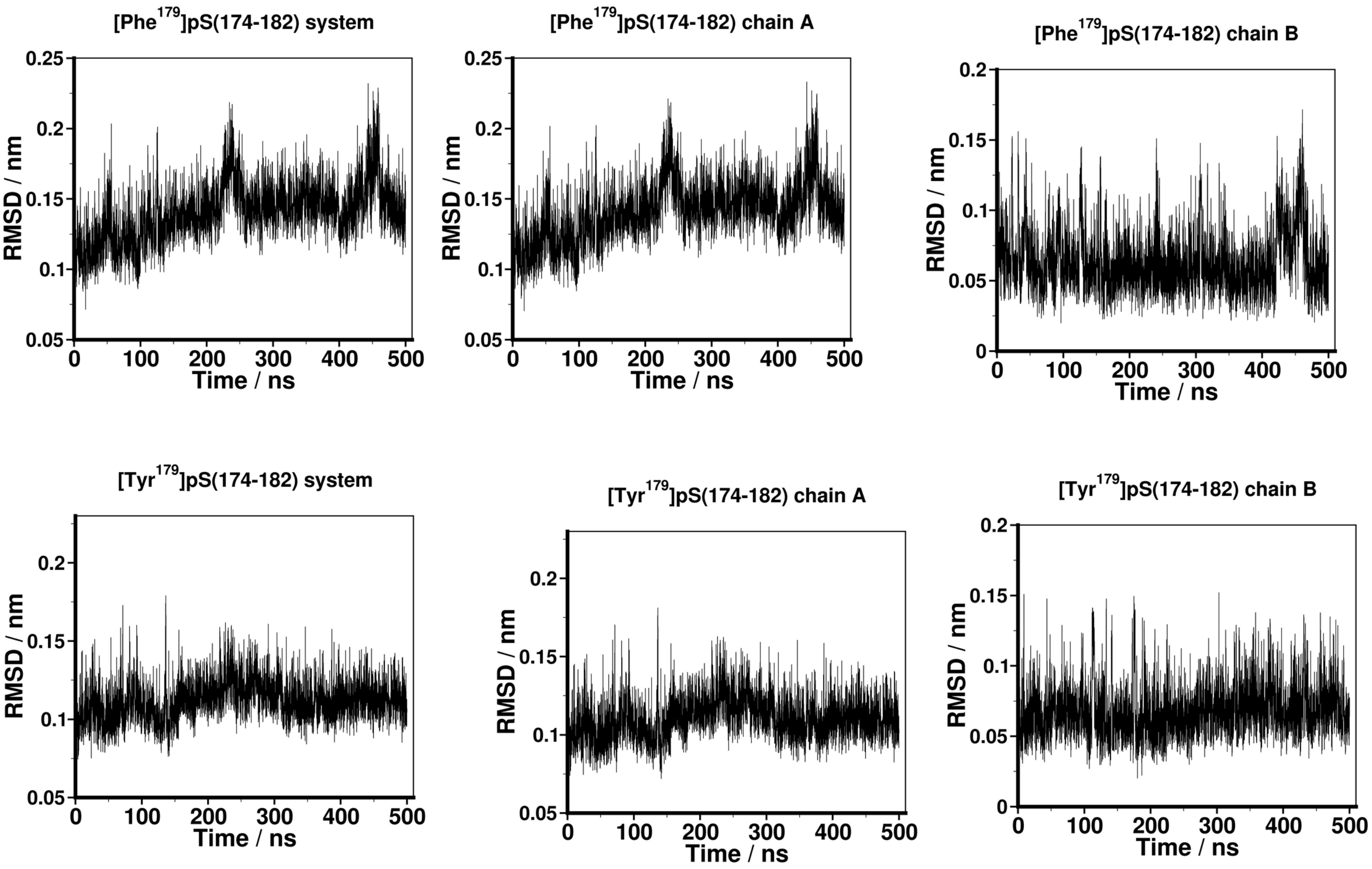

Figure S2. Molecular dynamics simulations of 14-3-3ε in complex with the 9 amino acid residue peptide analogs. System (14-3-3ε-peptide complex), chain A (14-3-3ε), chain B (peptide)

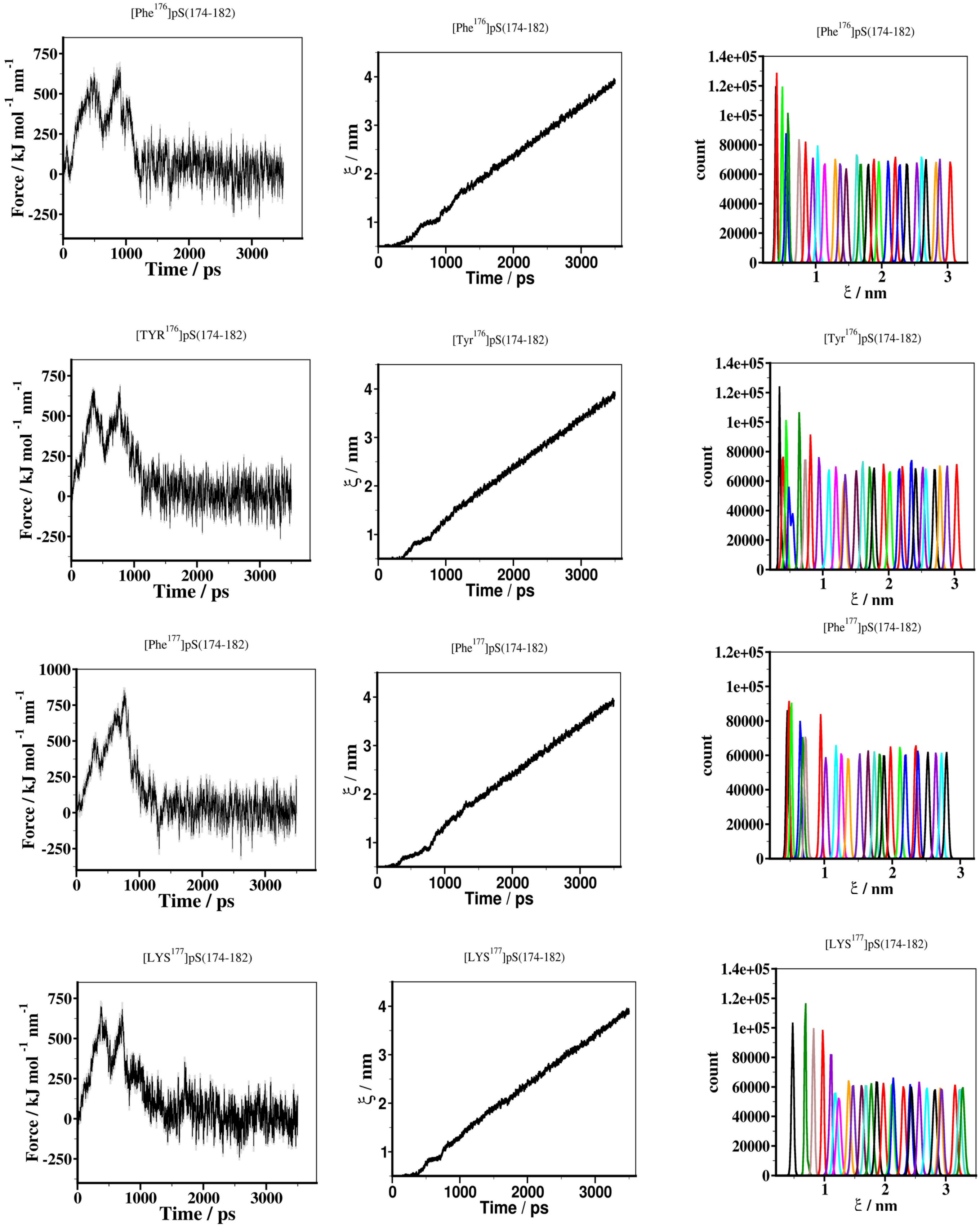

[Phe176]pS(174-182)
Force / kJ mol -1 nm -1
Time / ps
[Phe176]pS(174-182)
ξ / nm
Time / ps
[Phe176]pS(174-182)
count
ξ / nm
[TYR176]pS(174-182)
Force / kJ mol -1 nm -1
Time / ps
[Tyr176]pS(174-182)
ξ / nm
Time / ps
[Tyr176]pS(174-182)
count
ξ / nm
[Phe177]pS(174-182)
Force / kJ mol -1 nm -1
Time / ps
[Phe177]pS(174-182)
ξ / nm
Time / ps
[Phe177]pS(174-182)
count
ξ / nm
[LYS177]pS(174-182)
Force / kJ mol -1 nm -1
Time / ps
[LYS177]pS(174-182)
ξ / nm
Time / ps
[LYS177]pS(174-182)
count
ξ / nm

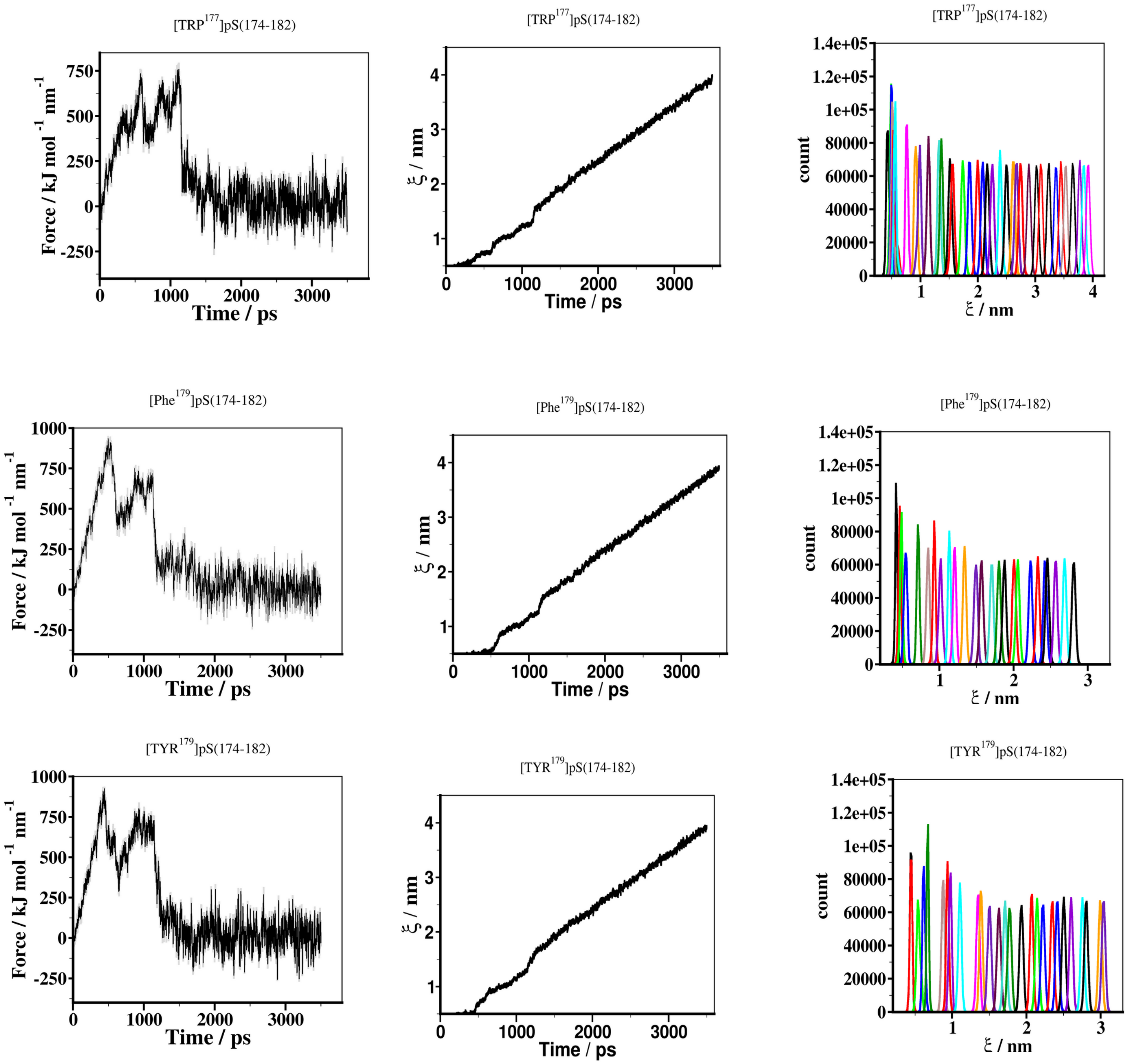

Figure S3. Steered MD simulation of 14-3-3ε – peptide complexes. External force applied to pull the peptide away from the protein during the simulation (left panels). Distance between COM of protein and COM of peptide during the simulation (middle panels). Umbrella sampling histograms of configurations from a 30 ns simulation (right panels)

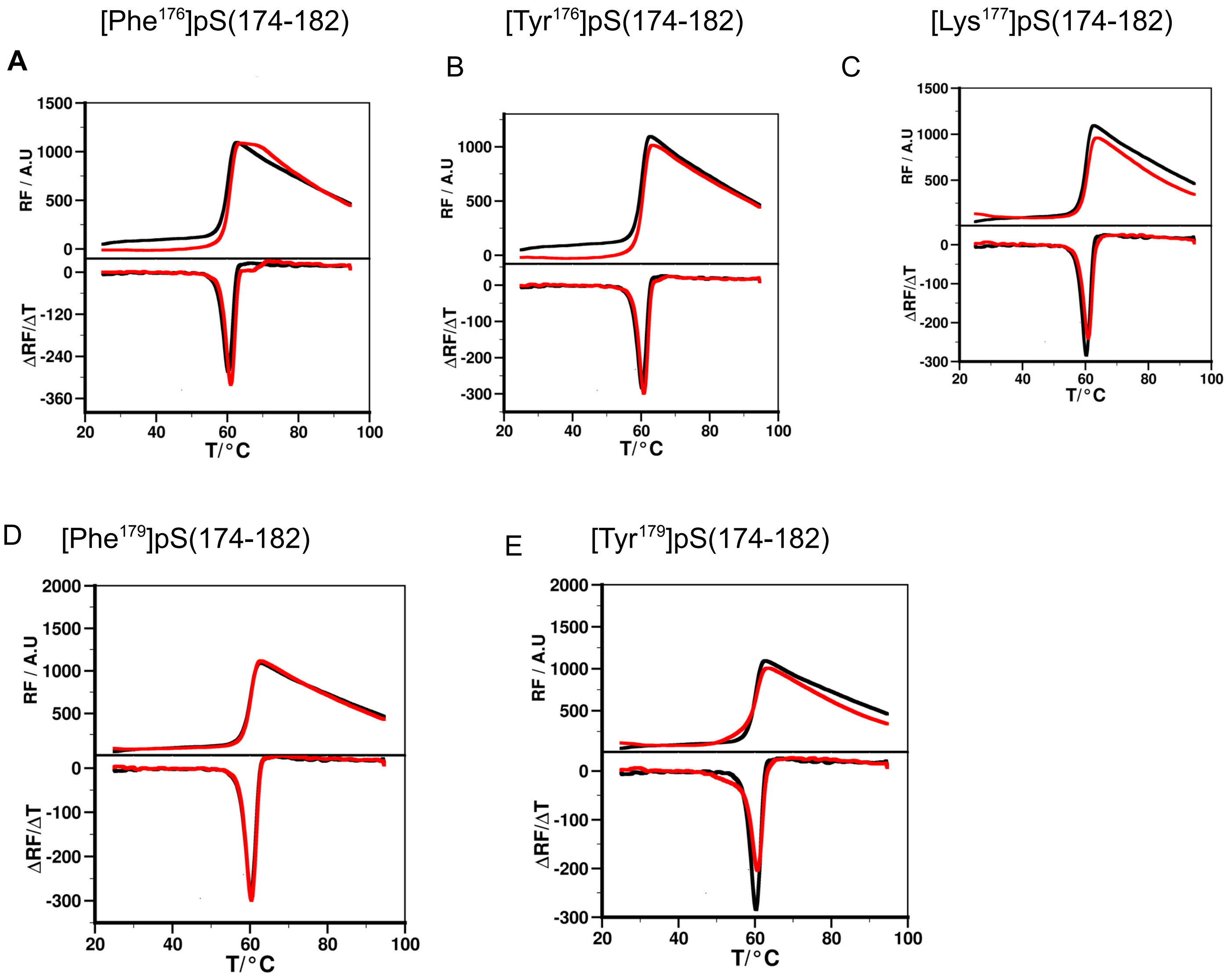

Figure S4. Thermal denaturation of 14-3-3ε studied using differential scanning fluorimetry. Melting curve of 14-3-3ε alone (black), and 14-3-3 in presence of a peptide (red), and their respective first derivative curve. (A) [Tyr^{176}] pS(174-182), (B) [Tyr^{176}] pS(174-182),(C) [Lys^{177}] pS(174-182), (D) [Phe^{179}] pS(174-182), (D) [Tyr^{179}] pS(174-182).

[Phe176]pS(174-182)

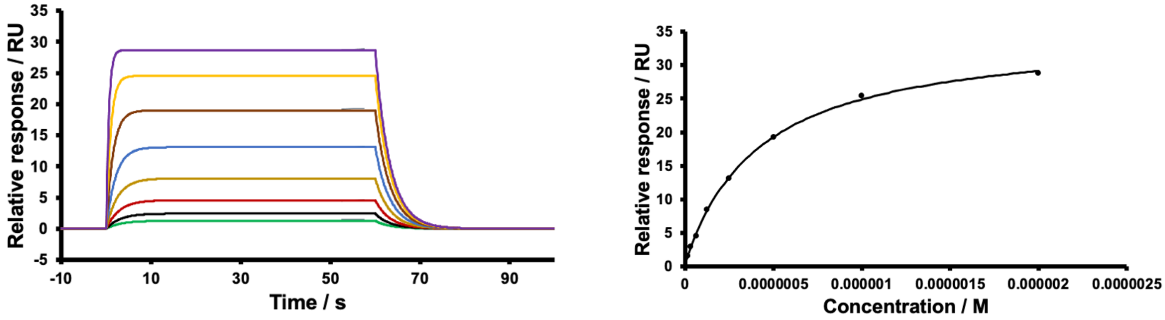

[Tyr176]pS(174-182)

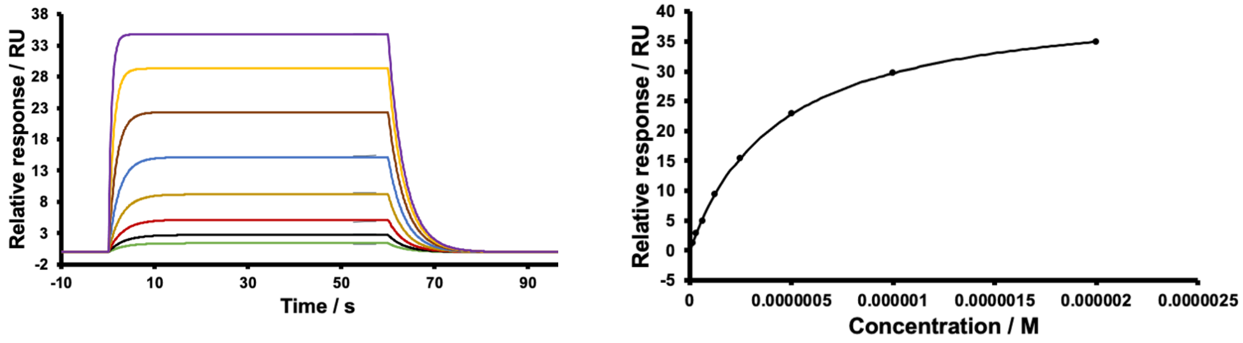

[Lys177]pS(174-182)

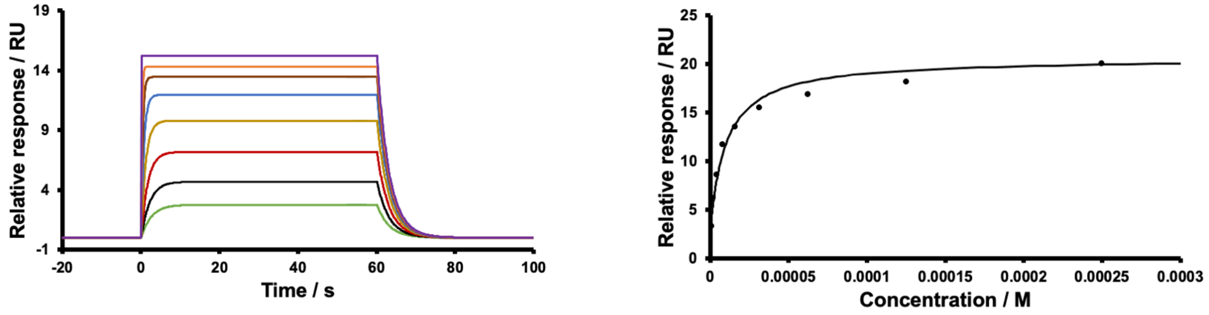

[Phe179]pS(174-182)

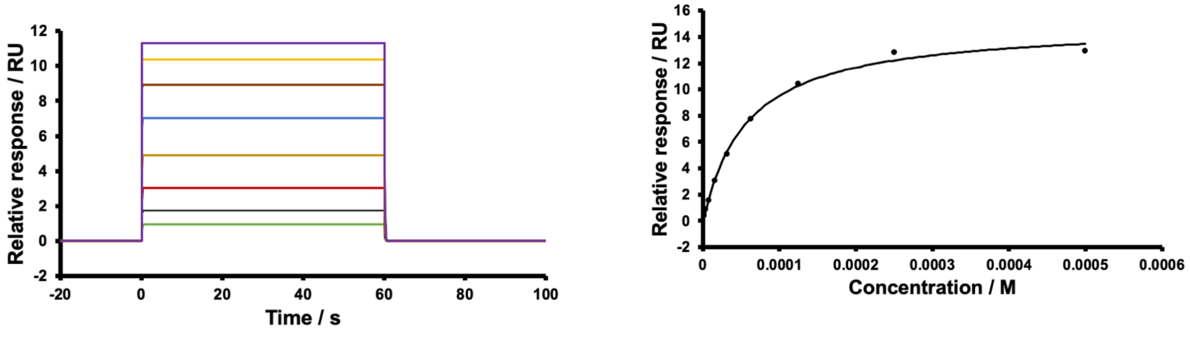

[Tyr^{179}]pS(174-182)

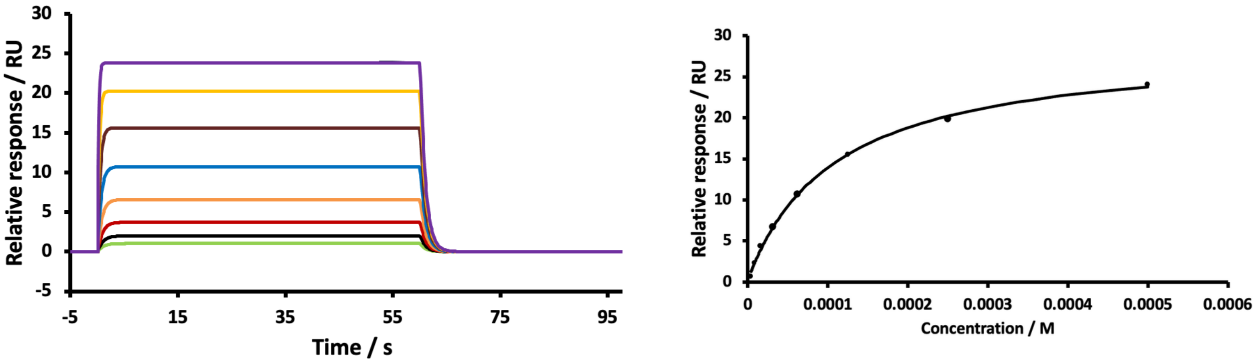

Figure S5: Representative results from surface plasmon resonance. 14-3-3ε was immobilized on NTA sensor chip. Binding of the ligands to 14-3-3ε was measured at concentration range of 0–100 μM. Sensograms (left panel) and their respective binding isotherm (right panel).

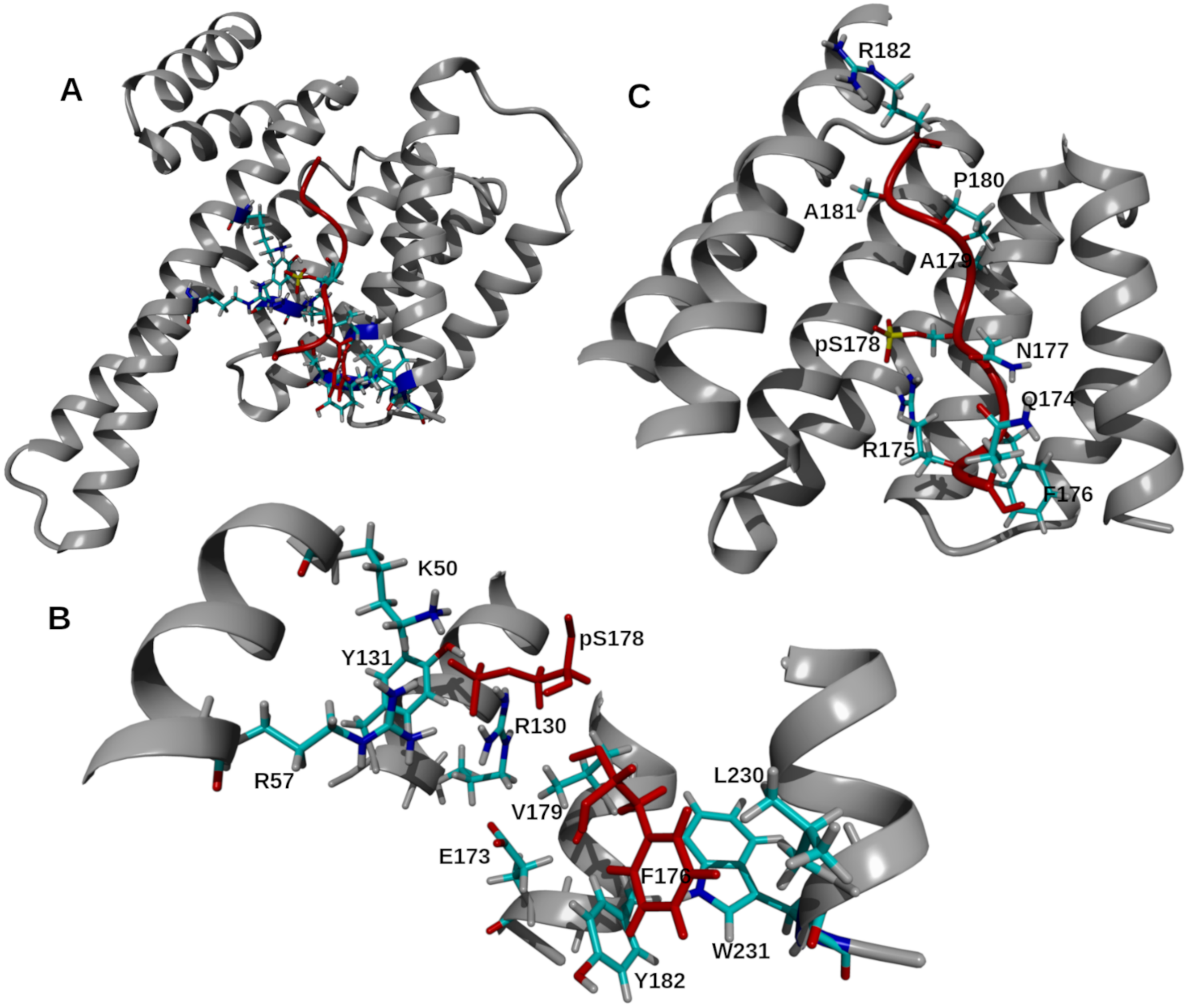

Figure S6: Interactions between 14-3-3ε protein and [Phe^{176}]pS(174-182) peptide
(**A**) The structures of 14-3-3ε - [Phe^{176}]pS(174-182) peptide complex, the structure of the protein and peptide are shown as gray ribbon and red tube, respectively. Atoms in pSer^{178} are reperesented as H, white; C, cyan; O, red; P, yellow stick. Phe^{176} is shown in red sticks. (**B**) A close-up view of (A) showing residues of 14-3-3ε that interact with pSer^{178} and Phe^{176} residues of the peptide. H-bonds were identified within 0.25 nm, hydrophobic and π-π interactions cutoff was 0.8 nm (**C**) A display of residues of the peptide in the binding groove of 14-3-3ε. Amino acid residues are labeled in one letter code in B and C.

Table S1. Interaction energies between the truncated peptides and 14-3-3ε

Peptide	Interaction energies (kJ/mol)
pS(173-185)	−1464.72 ± 134.06
pS(173-184)	−1422.65 ± 137.24
pS(173-183)	−1260.31 ± 32.18
pS(174-183)	−1251.05 ± 96.01
pS(174-182)	−1165.30 ± 128.29

Table S2. Components of ΔG of the peptide upon Ala-scan of the indicated residues

Residues	Polar	Non polar	Coulomb	Van der Waals	Polar solvation	Nonpolar solvation	-T ΔS	ΔG	ΔΔG
WT	-618.1	-213.6	-2073.0	-181.6	1454.9	-32.0	210.85	-620.83	NA
Gln174	-614.5	-213.3	-2066.0	-181.4	1451.5	-32.0	217.66	-610.24	10.59
Arg175	-544.2	-196.2	-1748.1	-165.1	1203.9	-31.1	238.62	-502.08	118.75
Gln176	-616.3	-191.7	-2034.0	-161.5	1417.7	-30.2	207.53	-600.46	20.37
Asn177	-602.1	-209.4	-2042.1	-177.6	1439.9	-31.8	205.93	-605.56	15.27
pSer178	429.4	-249.8	-1535.6	-219.0	1965.0	-30.8	218.90	398.50	1018.5
Arg182	-553.9	-201.9	-1073.0	-173.1	519.0	-28.8	161.20	-594.50	26.33

Energies are presented in kJ/mol. NA ; not applicable

Table S3. Ionic interactions between residues of pS(174-182) and 14-3-3ε during a 500 ns MD simulation

	Residues of 14-3-3ε	Residues of pS(174-182)	Occupancy (%)
**Saltbridges**	Glu134	Arg175	76.9
	Glu183	Arg175	83.9
	Asp216	Arg182	79.6
	Glu15	Arg182	20.2
	Glu40	Arg182	5.7
	Glu212	Arg182	5.01
	Asp226	Arg175	1.4

Table S4. Hydrogen bods between residues of pS(174-182) and 14-3-3ε, during a 500 ns simulation.

	Donor	Accepter	% occupancy
**H-bonds**	ARG130-Side	pSER178-Side	100.00
	TYR131-Side	pSER178-Side	99.18
	ARG57-Side	pSER178-Side	99.95
	ALA179-Main	ASN176-Side	96.52
	SLYS50-Side	pSER178-Side	95.72
	SER46-Side	ALA181-Main	70.60
	ASN227-Side	ASN177-Main	62.97
	ARG182-Side	ASN43-Side	61.64
	TRP231-Side	GLN176-Side	55.28
	ARG182-Side	ASP216-Side	44.84
	GLN176-Side	ASN227-Side	33.37
	pSER178-Side	ASN176-Side	27.58
	LEU175-Side	ASN177-Main	19.90
	ARG175-Side	ARG61-Side	15.28
	LEU230-Side	GLN176-Side	14.85
	LEU223-Side	ASN177-Side	13.05
	ASN177-Side	ASN227-Side	12.66
	LEU175-Side	ALA179-Side	11.91
	LEU175-Side	ALA179-Main	11.08
	GLN176-Side	GLU183-Side	11.03
	ALA181-Side	SER46-Main	10.97
	PRO180-Side	LEU223-Side	9.75
	PHE120-Side	ARG182-Side	9.08
	GLN176-Side	TYR182-Side	7.91
	ARG182-Side	ILE169-Side	7.73
	VAL179-Side	ASN177-Main	6.44

Residues of pS(174-182) are highlighted in red, and residues of 14-3-3ε are in black. Side (side chain); Main, (main chain).

Table S5. Composition of Secondary Structure of the pS(174-182)

Secondary structure	% of secondary structure
Random coil	77
β-bridge + β-turn	5
β-bent	19
β-turn	5

The secondary structure composition was calculated by SDDP method

Table S6. Free Energy of Binding of the Peptidessecond best

Peptides	ΔG_b / kJ mol^{-1}
pS(174-182)	-128.62 ± 8.05
[Phe^{176}]pS(174-182)	-148.76 ± 5.86
[Tyr^{176}]pS(174-182)	-157.23 ± 6.57
[Lys^{177}]pS(174-182)	-144.86 ± 7.04
[Phe^{177}]pS(174-182)	-132.15 ± 5.61
[Trp^{177}]pS(174-182)	-130.39 ± 5.43
[Phe^{179}]pS(174-182)	-140.96 ± 5.89
[Tyr^{179}]pS(174-182)	-145.79 ± 5.06

ΔG_b corresponds to the average and SD of n = 5.
The data is also presented in figure 4E.

Table S7. Composition of fraction of secondary structure of pS peptide analogs determined by ECD.

Peptide	% TFE	Helix	β-strand	β-turn	unordered	Total
pS	0	0.0	0.17	0.12	0.70	0.99
	30	0.17	0.15	0.13	0.55	1
	50	0.41	0.16	0.13	0.29	0.99
	70	0.5	0.12	0.14	0.24	1
pS(174-182)	0	0.0	0.09	0.06	0.84	0.99
	30	0.02	0.05	0.03	0.90	1
	50	0.02	0.07	0.04	0.86	0.99
	70	0.04	0.05	0.04	0.86	0.99
[Phe^{176}]pS(174-182)	0	0.02	0.16	0.11	0.69	0.98
	30	0.05	0.13	0.10	0.71	0.99
	50	0.05	0.18	0.10	0.66	0.99
	70	0.06	0.16	0.11	0.65	0.98
[Tyr^{176}]pS(174-182)	0	0.0	0.12	0.08	0.79	0.99
	30	0.01	0.14	0.08	0.76	0.99
	50	0.02	0.13	0.08	0.76	0.99
	70	0.04	0.14	0.08	0.74	1
[Phe^{179}]pS(174-182)	0	0.02	0.13	0.09	0.76	1
	30	0.02	0.17	0.10	0.69	0.98
	50	0.02	0.12	0.06	0.78	0.98
	70	0.02	0.11	0.06	0.79	0.98
[Tyr^{179}]pS(174-182)	0	0.02	0.28	0.15	0.54	0.98
	30	0.02	0.25	0.15	0.55	0.97
	50	0.01	0.30	0.17	0.50	0.98
	70	0.01	0.25	0.14	0.58	0.98

Components of secondary structures of peptides were obtained by deconvoluting ECD spectra using the CDSSTR method of DichroWeb web server.
